# A High Throughput Approach Based on Dynamic High Pressure for the Encapsulation of Active Compounds in Exosomes for Precision Medicine

**DOI:** 10.3390/ijms22189896

**Published:** 2021-09-13

**Authors:** Eugenia Romano, Paolo Antonio Netti, Enza Torino

**Affiliations:** 1 Department of Chemical, Materials Engineering & Industrial Production, University of Naples Federico II, Piazzale Tecchio 80, 80125 Naples, Italy; eugenia.romano@unina.it (E.R.); paoloantonio.netti@unina.it (P.A.N.); 2 Interdisciplinary Research Center on Biomaterials, CRIB, Piazzale Tecchio 80, 80125 Naples, Italy; 3 Center for Advanced Biomaterials for Health Care, CABHC, Istituto Italiano di Tecnologia, IIT@CRIB, Largo Barsanti e Matteucci 53, 80125 Naples, Italy

**Keywords:** extracellular vesicles, drug delivery, Glioma

## Abstract

In recent decades, endogenous nanocarrier-exosomes have received considerable scientific interest as drug delivery systems. The unique proteo-lipid architecture allows the crossing of various natural barriers and protects exosomes cargo from degradation in the bloodstream. However, the presence of this bilayer membrane as well as their endogenous content make loading of exogenous molecules challenging. In the present work, we will investigate how to promote the manipulation of vesicles curvature by a high-pressure microfluidic system as a ground-breaking method for exosomes encapsulation. Exosomes isolated from Uppsala 87 Malignant Glioma (U87-MG) cell culture media were characterized before and after the treatment with high-pressure homogenization. Once their structural and biological stability were validated, we applied this novel method for the encapsulation in the lipidic exosomal bilayer of the chemotherapeutic Irinotecan HCl Trihydrate-CPT 11. Finally, we performed in vitro preliminary test to validate the nanobiointeraction of exosomes, uptake mechanisms, and cytotoxic effect in cell culture model.

## 1. Introduction

Extracellular vesicles (EVs) are phospholipid-based vesicles released from the plasma membrane (PM) of many different cell types into almost all types of bodily fluids [1].

EVs play a critical role in both physiological and pathological conditions by their ability to participate in intercellular signaling and communication [2].

The three main subtypes of EVs include microvesicles (MVs), exosomes (Exos), and apoptotic bodies, which are sub-grouped on the basis of their biogenesis, release pathways, size, content, and function [3].

Among them, Exos are the most well studied group of EVs that present various advantages over traditional delivery vehicles [4,5,6]. Their intrinsic cell-targeting properties regulate the endogenous mechanism for intercellular communication [7,8]. However, Exos therapeutic applications as drug delivery systems have been limited due to a lack of efficient drug-loading methods [9]. To date, several approaches have been involved that can be summarized in two different processes, pre and post-isolation loading [10].

Generally, the loading of small molecules into Exos in a pre-isolation approach can be accomplished by transfection of the producer cell with the respective cargo by lipofection [11]. On the contrary, co-incubation and direct mixing can be performed both before isolation, generally at 37 °C combined with an apoptotic UV treatment, and after isolation.

Relatively successful results before isolation were obtained by Pascucci et al. [12]. They found that mesenchymal stem cells (MSCs) can acquire strong anti-tumor activity after long incubation with a high dosage of Paclitaxel (PTX). Wei et al. [13] recently explored the effect of mesenchymal Exos combined with doxorubicin (DOX) on osteosarcoma in vitro. After isolation, they found that the loading of Exos was achieved by mixing Exos and DOX with a final encapsulation efficiency (EE) of about 12%.

Although all these methods do not affect Exos integrity, there is a very low EE due to the lipid bilayer membrane, which restricts passive loading [14]. Moreover, hydrophilic drugs or vectors as nucleic acids cannot incorporate spontaneously into Exos since the drugs should cross the lipophilic Exos bilayer to reach the aqueous core.

Generally, post-isolation methods are preferred [15]. Recently, other active methods for loading Exos have involved several processes, such as electroporation, saponin treatment, sonication, extrusion or freeze-thaw cycles, and a combination of them [16]. Overall, saponin treatment and electroporation are the most useful methods to load Exos internally with hydrophilic cargo such as siRNA. Alvarez Erviti et al. showed that purified Exos were loaded with approximately 25% of exogenous GAPDH siRNA by electroporation [17]. Momen-Heravi et al. [18] showed that B cell-derived Exos could function as vehicles to deliver exogenous miRNA-155 mimic or inhibitor to primary mouse hepatocytes and RAW 264.7 macrophages, respectively. They optimized electroporation conditions in terms of voltage, capacitance, EVs concentration, and re-isolation method for effective miRNA-155 loading and recovery of B cell Exos. By setting optimizations, 55% of miRNA-155 mimic were loaded successfully into the Exos, obtaining encapsulation of small molecules in EVs with a quite high EE without affecting their integrity and function. However, it is a time-consuming and not reproducible procedure, inducing strong aggregation of siRNA even in the absence of Exos, leading to possible overestimation of the EE [19]. In addition, it might cause aggregation or fusion of Exos themselves [4].

Also, surfactants can be applied to generate pores on the exosomal surface, thus increasing membrane permeabilization. For example, saponin and triton have been used to dissolve membrane molecules (e.g., cholesterol) of cell membranes [20]. This combination significantly enhances the loading capacity of various types of molecules into Exos, like natural antioxidants [21] or chemotherapeutics [22]. In a recent study, DOX and PTX were successfully loaded into SF7761 stem cell-like GMs-derived Exos by a microfluidic device in the presence of saponin, applying shear stress in microfluidic channels [23]. EE of DOX and PTX was around 10%. However, there are issues regarding the in vivo hemolytic activity of saponin. Therefore, the concentration of saponin used for drug loading should be limited, and the Exos should be washed and purified immediately after co-incubation.

A valid alternative is represented by sonication. This physical strategy applies an extra mechanical shear force using a high gain ultrasonic probe [24]. The mechanical shear force compromises permanently and temporarily the membrane integrity of the Exos and allows the diffusion of drugs, proteins, or other nanomaterials inside the vesicles. The encapsulation by sonication of Exos is a spread-use loading method, especially for hydrophobic drugs. For example, a loading capacity of 11.68 ± 3.68% was demonstrated for Gemcitabine-loaded Exos treated with sonication compared to the incubated sample (2.79 ± 0.72%) [25]. Similarly, Salarpour et al. [26] compared the EE obtained by co-incubation and sonication, showing that the loading of PTX reached by sonication (9.21 ± 0.41 ngPTX/μgExos) was higher than co-incubation (7.40 ± 0.37 ng/μg). However, in some cases, drugs are not only encapsulated inside the Exos but also adsorbed to the outer layer. Furthermore, this method may lead to aggregation of nanovesicles and damage of their plasma membrane. Nevertheless, Kim et al. [27] demonstrated that this membrane deformation process does not significantly affect the membrane-bound proteins or the lipid contents of the Exos. Indeed, the membrane integrity at some conditions has been found to be restored within an hour after incubation at 37 °C. However, no information are reported about the composition.

Other methodologies for the encapsulation are based on extrusion. Generally, Exos and cargo mixture are forced into a lipid-based syringe under a controlled temperature. Repeated passages induce the vesicles membrane bilayer collapse and recombination with the drug [6]. Through this method, a slightly high loading efficiency (22.2 ± 3.1%) was achieved for catalase-loaded Exos [21]. Conversely, Fuhrmann et al. [28] reported that the loading efficiency of porphyrin into MDA-EVs was dramatically increased by co-incubation with 0.01% (*w*/*v*) saponin or by hypotonic dialysis, but not by extrusion. Furthermore, they found out that the extrusion method alters the Zeta Potential of the original Exos and causes cytotoxicity. So, whether the harsh mechanical force used in this method changes the membrane properties needs further investigation.

Moreover, extrusion-based strategy has also been explored to treat cells directly to induce the formation of Exos and offering an alluring prospect for the engineering of the so-called “vesicles-like” or “exosome-mimetic” structures [29]. In this case, cells could directly be treated with a chemotherapeutic through serial extrusion filters with diminishing pore sizes, thus allowing the production of Exos-Mimetic Nanovesicles (EMNVs) with an EE of 30% [30].

So together, all these reports demonstrate considerable advances and improvements for the delicate loading procedure. Nonetheless, there is no consensus on which technique results in being more advantageous, and there are still relevant obstacles to overcome before deploying EVs in large clinical trials. Indeed, the purification of Exos remains laborious, the integrity and biological activity is often compromised, and low EE is still a critical issue. Furthermore, up to date, the current processes have not addressed an industrial application of the Exos loading and provided a methodology that can guarantee a high and reliable EE in a cost-effective and scalable methodology, avoiding a time-consuming approach.

In our study, we propose high-pressure homogenization (HPH) as the methodology to improve the cargo loading of Exos. This HPH works in a turbulent flow situation, thus inducing very high shear forces on the fluid sample in the interaction chamber. However, to date, biological applications of HPH are only aimed at cell lysis for the extraction of proteins or lipids of interest [31,32,33].

Here, we analyze the dynamic of vesicles in turbulent flow that promotes mixing by a high-pressure system and takes advantage of this system to improve the loading of drug molecules within the Exos. Indeed, Exos, as all vesicle types, are a viscous droplet enclosed by a lipid bilayer. A classical lipid bilayer can be considered as a two-dimensional liquid with its membrane fluidity and mechanical properties [34].

So, the rationale behind this whole new encapsulation approach is to exploit pressure gradients along with both high shear and elongational stresses acting on vesicles to induce the formation of transient pores or permeabilization of Exos without disrupting them, thus allowing inward diffusion of cargos from the surrounding media.

Compared to other approaches, the goal has been to achieve the desired stability and reproducibility for loaded Exos treated by HPH. Moreover, applying this industrially scalable methodology allows obtaining a high EE in a very short process time and avoiding the use of permeabilization enhancers.

To validate our approach, we tested the prodrug Irinotecan (IRI). IRI is a chemotherapy agent used to treat a variety of solid tumors, such as colorectal, pancreatic, ovarian, and lung cancers, with promising activity against a broad spectrum of malignancies, including Glioblastoma Multiforme (GBM) [35]. It causes S-phase-specific cell killing by poisoning topoisomerase I in the cell and, as a prodrug, it needs to be converted in the active SN-38, a competitive analog of topoisomerase-I inhibitor [36]. To the best of our knowledge, no study on the interaction between IRI and Exos has yet been conducted to treat GBM. So, here the encapsulation of IRI by HPH in U87-derived Exos was investigated to test their Trojan horse’s function in the tumoral environment.

## 2. Results

### 2.1. Dynamic High-Pressure Homogenization Effect on Exos Stability

It is not well established to what extent the dynamic HPH allows systematic modification of morphology for different architectures. Therefore, an experimental campaign was conducted in Exos isolated by U87 cell line to evaluate their stability under dynamic HPH conditions. In detail, the process parameters that were intended to be fine-tuned are: pressure and cycles to ensure the structural stability of Exos; temperature, to avoid protein denaturation; and, moreover, dilution ratio, to prevent excessive material losses.

**Figure 1:**
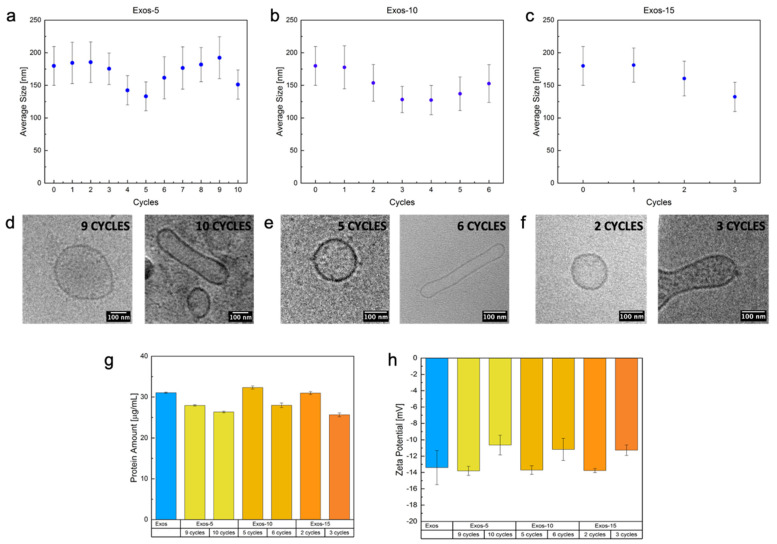
(**a**–**c**) Z-average size by DLS of (**a**) Exos-5 at cycles ranging from 0 to 10, (**b**) Exos-10 at cycles ranging from 0 to 6; and (**c**) Exos-15 at different cycles ranging from 0 to 3; (**d**–**f**) CRYO-TEM observations before and after the achievement of permanent deformation for relevant pressure value: (**d**) Exos-5 at cycles 9 and 10, (**e**) Exos-10 at cycles 5 and 6, (**f**) Exos-15 at cycles 2 and 3; (**g**) protein Concentration and (**h**) Zeta potential of untreated and treated Exos before and after the achievement of permanent deformation for relevant pressure values and cycles.

In Figure 1 are reported DLS results in terms of average size (Figure 1a–c) and morphology (Figure 1d–f) obtained at three pressure values, 500, 1000, and 1500 bar, by increasing the number of cycles up to 10.

The hydrodynamic radius (Rh) of Exos was calculated by DLS measurements. DLS data of untreated Exos (No HPH treatment) are also reported as control samples. The Z-average size of the control sample, obtained by DLS, was 170 nm with a polydispersity index (PDI) of 0.22 in line with literature [37,38,39]. In particular, PDI indicated a relative variability in particle size distribution for all the untreated Exos (Figure 1). SEM, TEM, Cryo-TEM Images, Particle Size Distribution (PSD), and NTA of untreated Exos (control sample) are reported in supporting info Figures S1 and S2.

In detail, Figure 1a–c reports the effect of the number of cycles on Exos’ average size at the three different constant pressures analyzed by DLS. In the samples treated at 500 bar (Exos-5), Figure 1a, three different phases were identified by increasing the number of cycles. In the first one, a constant average size was observed up to three cycles (phase 1), then a decrease in the range of three to five cycles (phase 2) was detected and, finally, size remained constant up to nine cycles (phase 3). After cycle 9, the size was not changing, but the instability and permanent changes in the morphology of the samples were observed (phase 4).

A similar phenomenon was also observed for Exos treated at 1000 bar (Exos-10), as shown in Figure 1b. However, at this condition, phase 1 at constant size was reduced to only one cycle while the average size of Exos promptly decreased between two and four Cycles (phase 2) and remained constant up to six Cycles (phase 3). After six cycles, however, instability and changes in morphologies were again detected (phase 4). Finally, for Exos treated at 1500 bar (Exos-15), a shortening of the phases was reported (Figure 1c). Indeed, a similar prompt reduction was observed up to two cycles (phase 2) but also direct transition to phase 4 was detected. DLS results obtained at numbers of cycles beyond phase 4 for all the samples are not reported due to the measurement’s instability.

In Figure 1d–f, CRYO-TEM images of the Exos corresponding to the identified phases are reported at the different conditions. The images were used to evaluate the link among size, at different pressures and cycles, and coalescence, breaking, and deformation phenomena affecting the Exos membrane and stability. A further explanation of these phenomena will be reported in the Section 3.

As shown in Figure 1d, even though DLS reported a decrease in size, Exos-5′morphology remains spherical up to nine cycles while, after nine cycles, elongated Exos-5 are detected. Starting with these observations, we can hypothesize the overlapping of several phenomena in the phases. As previously reported on liposomes and oil-in-water emulsions treated by HPH [40,41,42,43,44,45], we may suppose that in the first phase, shear forces cut the bilayer membrane only of larger vesicles, reducing the polydispersity and, therefore, the principal peak value of PSD. Moreover, we have to consider that a small number of amphiphiles in aqueous solution could re-assembly to reduce their thermodynamic energy, forming smaller bilayer structures without compromising the role of naive Exos. However, in the second phase, the nanovesicles seem to be subjected to a temporary deformation that becomes permanent only at 10 cycles.

The same phenomena of break up and size reduction seemed visible also at 1000 bar, Exos-10, where the smallest particles and reassembled vesicles in turbulent flow are probably subjected to a dynamic deformation of lipidic bilayer that becomes permanent only at six cycles (Figure 1d—left). Moreover, it was possible to notice in both Exos-10 and Exos-15 an almost complete overlap of average size behavior up to three cycles. Nevertheless, Figure 1f—left demonstrated a combination of size decrease and permanent elongation of the particle membrane. So, for Exos-15, we may hypothesize that overlapping of breaking and permanent deformation phenomena is occurring. In such dynamic high-pressure conditions, they directly become non-convex, thus maintaining the characteristic minimum energy shape in a quiescent flow. In this situation, the vesicle membrane serves as a geometrical constraint maintaining the surface area and the enclosed volume conservation.

Finally, the conducted experiments highlighted that morphology of Exos drastically changed while approaching phase 4, where a permanent deformation induced by the combination of pressure and the number of cycles is observed for all the treatments. Furthermore, it was possible to identify for each pressure a specific cycle leading to breaking, coalescence, or permanent deformation of lipidic bilayers.

To assess the surface properties and biological stability of Exos-5, Exos-10, and Exos-15 just before and after the transition between phase 3 and 4, we analyzed protein content and Zeta potential in comparison with untreated vesicles. Results are reported in Figure 1g–h for the protein content and surface charge, respectively.

BCA assay was performed to confirm the total protein content and the absence of protein denaturation and degradation phenomena, possibly due to shear and elongational forces and high temperatures encountered in HPH valve (Figure 1g). Protein denaturation can be defined as an alteration in the biological, chemical, and physical properties of the protein by mild disruption of its structure [46]. It consists of the irreversible loss of the three-dimensional structure due to the breakage of non-covalent bonds brought by using various chemical denaturants or changing temperature or pH [46]. Most bio-functional proteins, such as enzymes with specific bioactivities, are susceptible to denaturation; even slight changes may cause inactivation and result in the loss of their biological function [47]. In our case, a stable amount of surface proteins with respect to the control is reported for all the conditions at phase 1, 2, and 3. However, only at phase 4, a reduction from 10 to 30% of total protein content is observed when the permanent deformation is achieved. We believe that the stability of protein at high pressure and cycle is supported by the operative choice to perform the process under mild pressure conditions keeping the temperature in a range of 5–10 °C, as reported by several studies in different fields [31,48,49,50].

Similar behavior is also reported by Zeta potential analysis in Figure 1h for all the samples. Indeed, it has already been proved that Exos spontaneously acquires a surface electric charge when brought into contact with a polar medium, such as a PBS buffer, and, like the plasma membrane of cells, their surface is generally negatively charged [51]. Our results on untreated and treated Exos up to phase 3 also showed a surface charge of about −15 eV. However, we detected a reduction of up to 30% of the surface charge only at phase 4. We can hypothesize that, for all the treated Exos approaching phase 4, this reduction can be attributed to the partial denaturation of protein, aggregation, and breaking of the Exos. Moreover, also the deformation altering the charge distribution and detection on the surface can occur, thus interfering with the stability of the colloidal system of Exos and reflecting in a less negative Zeta Potential value [51].

### 2.2. Encapsulation by HPH of Irinotecan in U87-Derived Exosomes

**Figure 2:**
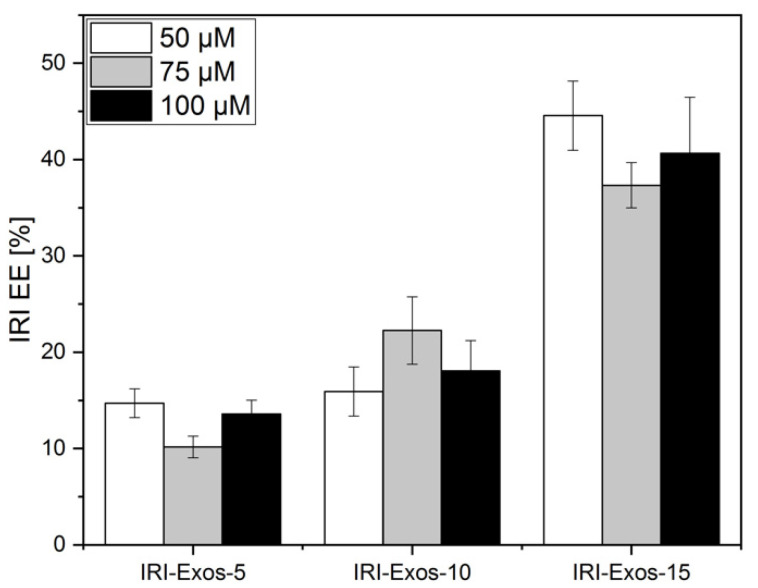
EE% IRI-Exos treated at gold standard conditions Exos-5, Exos-10, and Exos-15, at different IRI concentrations of 50, 75, and 100 µM.

Here, we present how our studies on the effect of HPH on the Exos’ morphology can be utilized to control the drug-loading capacity of bilayer natural vesicles (Figure 2). In the previous paragraph, we reported that Exos treated by HPH undergoes a temporary or permanent deformation of the membrane. We also proved that the temporary deformation happens without destabilizing their proteo-lipidic architecture, surface charge, and total protein cargo. Therefore, we selected the condition at which the temporary deformation occurs to perform all the following experiments. In detail, in the next experiments, we will always refer to Exos-5 at nine cycles, as *Exos-5*, Exos-10 at five cycles as *Exos-10*, and Exos-15 at two cycles as *Exos-15*, and will refer to them as “gold standard conditions”. Figure 2 reports EE% of the HPH approach for Exos treated at standard conditions Exos-5, 10, and 15, starting at three different theoretical concentrations of IRI. In detail, the same amount of Exos was added to a water solution containing 50, 75, and 100 µM of IRI (*IRI-Exos*). The obtained suspensions were treated at the standard conditions Exos-5, Exos-10, and Exos-15. Results showed that the entrapment of chemotherapeutic agent was promoted through a pressure-dependent manner: the higher the pressure value, the higher EE%, increasing from about 15 to 40% at different pressures by keeping the IRI concentration constant. In particular, for the condition of IRI-Exos-15, a stable morphology (Figure S4) and a stable and average value of EE of 40% was reported at all the IRI concentrations. The EE results of co-incubated Exos obtained at different IRI concentrations and treated at 37 °C for 2 h are reported in the Supporting Materials, Figure S3A, and showed a constant EE of about 8% for all the IRI concentrations.

As already stated, therapeutic agents were encapsulated into Exos using various passive and active methods, including co-incubation at room temperature with or without saponin permeabilization, electroporation, freeze-thaw cycles, sonication, extrusion, and dialysis [28,52]. Usually, as previously described, these methodologies for the encapsulation of drugs in Exos are time-consuming, resulting in poor stabilities of the drugs in Exos and a variable and very low loading efficiency up to 10%. Up to date, no similar EE has ever been achieved with other encapsulation methods and, moreover, obtained in a fast, highly effective, easy to make, and reproducible process that could really change the application of Exos in the treatment of diseases. Indeed, the proposed approach can allow the stable encapsulation also of low-soluble drugs, such as IRI, also used for GBM [53], and pave the way to their repositioning on other diseases, such as brain tumors, where their pharmacodynamics can be effective, but they are rejected due to their poor delivery properties.

### 2.3. Drug Release Behavior and Biological Interactions of Exos-15

#### 2.3.1. The exploitation of stability, release behavior of Exos-15 with Irinotecan (IRI Exos-15)

**Figure 3:**
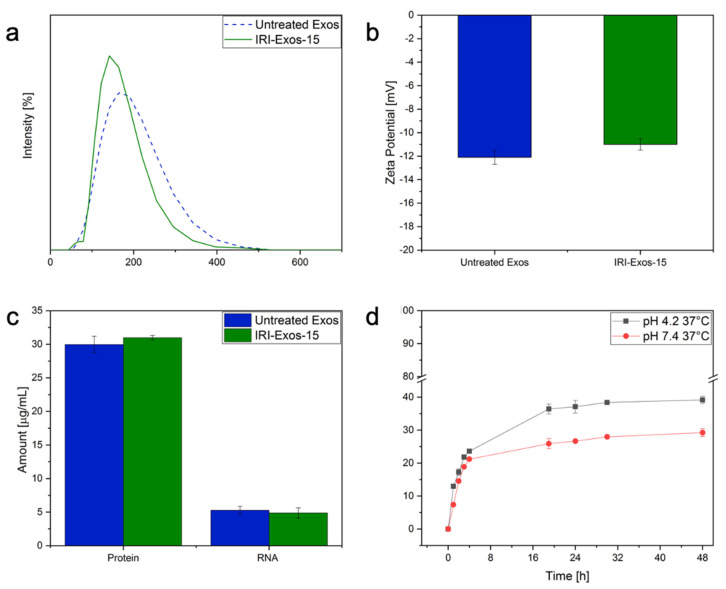
A comparison between untreated Exos and IRI-Exos-15: (**a**) PSD by DLS, (**b**) Zeta potential, (**c**) surface protein and RNA amount; (**d**) drug release profile of IRI-Exos-15 up to 48 h in PBS at 37 °C and pH 4.2 and pH 7.4.

To evaluate the cargo ability, release behavior, and biological interactions of IRI-Exos-15 obtained with HPH with respect to the traditional encapsulation by co-incubation, we studied their release profile, surface charge, and biological specificity by DLS, Zeta potential, surface protein and RNA amount. (Figure 3a–d).

In particular, we investigated the nano-bio interactions and drug delivery ability for IRI-Exos-15. IRI-Exos-15 was selected because of the higher achieved EE allowing to evaluate the effect of the IRI therapeutic dosage between 1 and 100 µM [51]. DLS and Zeta potential results in Figure 3a,b show a regular particles size distribution and also stable surface charge, respectively, compared to untreated Exos. Properties of untreated IRI-Exos obtained by co-incubation are reported in Figure S3b. Also, the surface protein analysis and RNA extraction after vesicles lysis (Figure 3c) further demonstrated that the encapsulation did not affect the transport of their naive biological content in the outer and inner side of the lipidic layer.

Finally, to investigate the in-vitro drug release profile of IRI-Exos-15, we mimicked the physiological environment and endolysosomal compartment respectively at pH of 7.4 or 4.2, 37 °C, up to 48 h (Figure 3d). In PBS dialysate at pH 7.4, the release of Exo-IRI was about 25% after 24 h, while in PBS dialysate at pH 4.2 the release rate achieved was about 40%. This result confirmed that an acidic environment, such as late endosomes and lysosomes of cancer cells, accelerates its release and its activation. The observed behavior can be considered relevant in the ability of Exos to escape the intracellular barriers, allowing the release, activation, and pharmacodynamics of the drug, and, therefore, in our case, the esterification of IRI prodrug in its metabolite SN-38 [52].

#### 2.3.2. Cytotoxicity, Cytofluorimetry Study and Uptake Behavior of Exos-15 with U87 Cells

**Figure 4:**
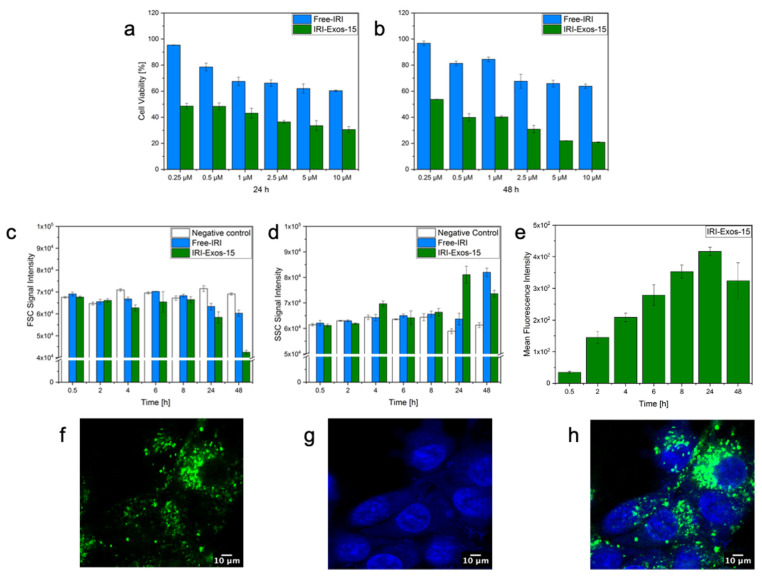
MTT Cytotoxicity assay of IRI-Exos-15 and Free-IRI cultured on U87-MG cells at 24 h (**a**) and 48 h (**b**); Flow cytometry of IRI-Exos-15 up to 48 h of incubation with U87-MG cells: (**c**) FSC signal, (**d**) SSC analysis, and (**e**) mean fluorescence intensity. All data are presented as mean ± s.d (*n* = 3). Images of Exos internalization at 24 h of incubation with U87-MG cells; (**f**) IRI-Exos-15 stained with PKH67; (**g**) U87-MG cells nuclei stained with Hoechst; and (**h**) merged channels.

According to the literature, free-IRI shows benefits in attaining lower cell viability or, equivalently, higher cytotoxicity at maximum concentration of 100 μM [54,55]. As IRI inhibited cell proliferation mainly by arresting cells in the G2-S phases of the cell cycle, in the present study, IRI-Exos-15 in vitro behavior on U87 cells was recorded up to 48 h to allow complete cells proliferation. Cytotoxicity analysis (Figure 4a,b) was performed, ranging the IRI concentration from 0.25 to 10 μM. Cell viability decreased for both Free-IRI and IRI-Exos-15 after 24 and 48 h, reaching the minimum viability of 70% and 20%, respectively, in 48 h. Results confirmed the enhanced cargo ability of IRI Exos-15 in terms of faster uptake and improved cytotoxicity at lower concentration with respect to the free-IRI, holding great promise to look at Exos as a safe and efficient means for precision medicine.

Furthermore, to evaluate the interaction between cells and IRI-Exos-15, quantitative measurement of nanoparticle uptake by flow cytometry was performed (Figure 4c–e). Forward scatter (FSC) is a parameter representative of cell size. Changes in FSC intensity reflect the swelling or shrinking of cells [56] and indirectly indicate cell viability [57]. IRI-Exos-15 and free-IRI were tested at 10 μM up to 48 h. Figure 4c showed a slight reduction of cell viability after 24 h for both IRI samples compared to negative control. However, as expected, IRI-Exos-15 at 48 h had higher cytotoxicity if compared to free-IRI. This data was also confirmed by side-scattered light (SSC) intensity (Figure 4d). Indeed, SSC is related to the number and type of organelles present in the cell [58]. This inner granularity value has often been used to show differences in the physical state of the cell, including mitosis and particle uptake [56,59].

Interestingly in our case, SSC increases just at 24 h up to 48 h. As a confirmation, also fluorescence intensity mean, reported as internalization value in Figure 4e, showed an increasing trend with a maximum value up to 24 h of cell coincubation. However, the signal remains high also up to 48 h, highlighting a competition between the number of cells replicating and the amount of treated Exos available in the system that could hide the cytotoxic and internalization phenomena.

Taking together all these considerations, we can speculate that at 24 h, we reached the maximum peak of internalization of vesicles inside cells. This uptake produces an increase in granulometry and a decrease in the size of tumoral cells, leading to the cytotoxic activity of IRI.

Furthermore, the ability of cells to internalize Exos was assessed with confocal microscopy (Figure 4f–h). Nanovesicles were distributed throughout the cytoplasm, especially in the perinuclear region. Moreover, the brightest signals were observed after 24 h of incubation, confirming cytofluorimetric analysis.

## 3. Discussion

Exos are small endosomal-derived membrane nanovesicles that have observed increasing attention over the past decade as a novel model of intercellular communication, impacting many cellular processes, such as signaling, antigen presentation and T cell stimulation, and immune response.

Moreover, only recently, these EVs have been investigated for precision medicine as functional vehicles that carry an endogenous and exogenous cargo of proteins, lipids, and nucleic acids, capable of delivering these cargos to specific target cells. However, the use of their promising properties in the nanobiotheranostic field to deliver active compounds such as tracers or drugs has been limited by the lack of efficient drug-loading methods [10].

In the current study, we propose HPH to improve the cargo loading of Exos. To date, HPH has been applied as an efficient means of cellular disruption extracting intracellular products such as DNA, ribosomes, and mitochondria; moreover, it has been industrially used in the food engineering field.

Currently, methodologies used for the encapsulation of Exos obtain an EE maximum value of 10%, while the use of HPH for the treatment of Exos that we propose allows for the first time an increase of the EE up to 45%, reaching the best values of EE obtained for the traditional encapsulation of nanoparticles, and making the cargo properties of Exos comparable with polymer and lipid-based vectors and valuable in the precision medicine field.

In our procedure, Exos samples are subjected to dynamic high pressures and are forced through a narrow static valve, undergoing stress forces, such as cavitation, and shear is generated. In detail, the operative parameters such as the valve geometry, pressure level, inlet temperature, and the number of homogenization cycles are analyzed. They affect the thermodynamic of the lipid bilayer by controlling turbulence, high shear, cavitation, and temperature, enhancing the stability of Exos, stabilizing the surface protein, and increasing the loading of active compounds.

It was already reported [60] for the bilayer of living cells that various kinds of environmental stress, such as temperature stress and osmotic stress, cause alterations in the physical properties of the membrane lipids due to the presence of proteins and cholesterol on their surface.

Here, the above HPH parameters were investigated to tune the loading capability of the Exos, taking advantage of the fluidity of their bilayer membrane. Indeed, as previously reported [61], cholesterol and proteins are also essential components of exosomal membranes and, among the biomolecules, are responsible for the membrane fluidity. Furthermore, they are most sensitive to high pressure. It is well-known that specific lipids are enriched in Exos compared to their parent cells and that lipid class enrichment in EVs depends on vesicle type and source cell type [62]. Our results started from U87, originating by Human glioblastoma astrocytoma, for the isolation of Exos. From literature, it is reported that Glycolipid, free fatty acid, and phosphatidylserine enrichment is generally observed in all U87, mainly used in this work, while Lyso derivatives and structural membrane lipid are usually depleted in Exos [60]. Therefore, the role of these lipid components could also interfere with the fluidity of the membrane and, therefore, the stiffness and flexibility, influencing the thermodynamic state under viscous stresses at high-pressure conditions.

Furthermore, the effects induced by the temperature have to be necessarily taken into account in HPH, where the high velocity of the fluid flow, which is then impinging on the zirconium valve of the homogenizer, leads to the dissipation of a significant fraction of the mechanical energy as heat in the fluid temperature increase. Indeed, during homogenization, a rise of the temperature (about 2.5 °C per 10 MPa), related to the fluid employed, is generally observed in the fluid downstream of the valve. We decide not to take advantage of the high temperature to protect the stability of the proteins. However, at low temperatures, phospholipids tend to cluster together, but steroids in the phospholipid bilayer fill in between the phospholipids, disrupting their intermolecular interactions and increasing fluidity. Therefore, we took advantage of this low temperature-sensitivity behavior of the intracellular components, and it was crucial to control and limit the product exposure to temperature increase by a cooling jacket at 10 °C at the inlet of the instrument and through heat-exchanging cooling coils to extract heat quickly, and nullify the temperature increase, affecting the permeabilization only by the high-pressure effect.

So, in the current study, we successfully improved and shortened to less than 1 h the encapsulation process of the Exos through HPH, preserving their morphological integrity and biological identity, and repositioning the Exos in the first line for the exploitation of their use in the nanobiotheranostic field.

Moreover, all the operative parameters were tested and threshold conditions in terms of pressure and number of cycles were identified with respect to the stability of the Exosomes.

To validate our approach, we selected the prodrug Irinotecan (IRI) currently under investigation for the treatment of GBM due to its ability for cytotoxic activity against central nervous system tumor xenografts and glioblastoma cells with multi-drug resistance [63]. The operative parameters, pressure and number of cycles were studied to control the EE % of the HPH approach for Exos treated at constant temperature.

Three different theoretical concentrations of IRI were tested at three different pressures and selected number of Cycles that had not preliminary showed instability of Exosomes or protein denaturation. A relevant and constant EE of 45% was reported at Exos-15 (Exos treated at 1500 bars and two cycles), obtained in about 45 min, which is much shorter than the usual methodology several hours or days longer with an ineffective EE of about 10%. Exos-15 was, then, considered as the gold standard condition for all the other experiments. We also investigated the cargo ability, release behavior, and biological interactions of the IRI- Exos-15, comparing them with traditional encapsulation by co-incubation. The synthetic identity of the Exos, such as their release profile, surface charge, and biological specificity, were studied by DLS, Zeta Potential, and surface protein and RNA amount, and results showed a constant release behavior of IRI Exos-15 at 48 h. Furthermore, in vitro tests on U87 cells, obtained by flow cytometry and confocal microscopy, proved the effect of their biological identity, showing a maximum peak of internalization of vesicles inside cells at 24 h and higher cytotoxicity at 48 h with respect to the free-IRI.

In conclusion, we established a feasible approach based on HPH to improve and shorten the loading of Exos at effective therapeutic concentrations. Indeed, as already stated, up to date, the current application and translation of Exos to clinics were limited by a series of key factors, among them the stable and adequate loading methods, that should be over crossed. From this perspective, our results confirm Exos’ high sensitivity and specificity that remain ideal candidates for early diagnosis and effective therapy. We proved that an approach based on high-pressure homogenization could potentially speed up their translation in the clinical practice, being a repeatable process guaranteed to scale up to pilot and/or production volumes.

## 4. Materials and Methods

The Human glioblastoma cell line U87 (passages 15–28) was purchased from ATCC. The cell line was cultured in DMEM supplemented with 10% foetal bovine serum (FBS), 0.1% penicillin–streptomycin and 0.1% L-glutamine (growth medium). All media and reagents for cell culture were purchased by Sigma Aldrich (St. Louis, MO, USA) and all cells were incubated at 37 °C in 5% CO_2_/95% air.

Cell viability was assessed by trypan blue stain 0.4% (Invitrogen^TM^, ThermoFisher Scientific; Waltham, MA, USA) and determined using a Countess II FL Automated Cell Counter (ThermoFisher Scientific; Waltham, MA, USA).

Irinotecan HCl Trihydrate-CPT 11 (molecular formula C_33_H_39_ClN_4_O_6_, *M*_W_ = 677.18 g/mol) was purchased from Selleckchem (Houston, TX, USA).

### 4.1. Exos Production and Isolation

4 × 10^6^ cells per 150T culture Flask were seeded and left to grow until 70–80% confluency. At this stage, adherent cells were washed in PBS (1x) twice, the culture media (CM) was removed and replaced with complete CM containing exosome-depleted foetal bovine serum growth (ThermoFisher Scientific, Waltham, MA, USA). After 48 h incubation, 50 mL CM was collected under sterile conditions and transferred to 50 mL polypropylene centrifuge tubes for Exos isolation. 

Exos were isolated by differential ultracentrifugation (dUC). Cell culture supernatants were harvested and centrifuged for 10 min at 300× *g* (F15-6x 100y ROTOR) using a SL 16R Centrifuge to get rid of dead cells, then at 2000× *g* for 10 min and other 30 min at 10,000× *g* to eliminate cell debris. Exos were pelleted by dUC of supernatant in 8 mL polycarbonate tubes (Beckman Coulter, Brea, CA, USA) at ~110,000× *g* (70,000 RPM–MLA-80 ROTOR) for 70 min using an Optima^TM^ MAX-XP Ultracentrifuge (Beckman Coulter, Brea, CA, USA) and the supernatant was discarded. Finally, pellet resuspended in PBS was washed twice. All procedures were carried out at 4 °C. The purified Exos were resuspended in 200 μL of PBS and stored at −20 °C prior to use.

### 4.2. Exos Treatment by High Pressure Homogenization

To assess the stability of Exos subjected to HPH, samples were processed using a bench-top microfluidizer (Model M-110P Microfluidizer^TM^ Materials Processor, Microfluidics, Westwood, MA, USA) (94 × 71 × 56 mm, w × d × h). To evaluate the effects of the pressures and cycles, three different points at 500-1000-1500 bar and from 1 up to 10 cycles were tested. The microfluidic device divides the suspension feed into two opposing microchannels in a Y fixed-geometry interaction chamber (diamond F20 Y-75 μm chamber). Then the two jets of liquid suspension are forced to collide with each other at high pressure, creating extreme shears, along with cavitation and impact. 

A thermocouple was placed in the reservoir close to the discharge port to monitor temperature fluctuations during HPH. An ice bath to cool the cooling external coil was used to keep the temperature in a range from 4–10 °C.

### 4.3. Morphological Characterization and Biological Identity of U87 Cell-Derived Exosomes

Size, morphology and purity of Exos were observed before and after HPH treatment to validate the stability of vesicles.

For Cryo-TEM analysis, 3 μL of sample were directly dropped onto Formvar/Carbon 200 mesh Cu Agar^®^ grids. Samples were observed in a Tecnai FEI^®^ TEM operated at 80 kV accelerating voltage.

The concentration and mean size of Exos were determined by recording and analyzing the Brownian motion of particles using a NanoSight NS300 system and NPs Tracking Analysis (NTA) 3.3-Sample Assistant Dev Build 3.3.203—Analytical Software (Malvern, Worcestershire, UK) according to the manufacturer’s protocol. Purified Exos were diluted 1:100 in 1 mL PBS at RT and monitored for 260 s with manual shutter and gain adjustments. The recorded videos were analyzed using a NPs Tracking Analysis software. Mean size of Exos diluted in water (0.8% wt/vol) was analyzed at 25 °C, using a Zetasizer Nano ZS (Model ZEN3600, Malvern Instruments Ltd., UK) equipped with a solid-state laser (λ = 633 nm) at a scattering angle of 173°. The cuvettes used are the 12-mm square glass cuvettes with square aperture for 90° sizing (Malvern; # PCS1115). The recovery of Exos was indirectly estimated by measuring the surface protein quantitation using the BCA assay. Total protein amount was quantified with QuantiPro^TM^ BCA Assay Kit (Sigma Aldrich; St. Louis, MO, USA) in each vesicle preparation.

### 4.4. Entrapment of Irinotecan in Exosomes by High Pressure Homogenization

IRI was incorporated into Exos through HPH. For high drug-loading efficiency, a series of ratios were assessed. Three different molar concentrations (50-75-100 μM) were pre-mixed with naive Exos at a fixed concentration of 3.24 × 10^23^ particles/mL in PBS and fed to the system through a stainless-steel feed reservoir. HPH was carried out at fixed pressures and cycles (500 bar—nine cycles, 1000 bar—5 cycles, 1500 bar—two cycles). Then, the collected sample was purified through Spin-X corning Centrifugation 3KDa Cut-off performed at 3000× *g* 60 min 4 °C.

Finally, EE was determined. The Exos bilayer was disrupted with 0.075% *v*/*v* Triton X-100 to release the encapsulated drug. The concentration of drug in the solution was determined using a UV−vis spectrophotometer at a wavelength of 364 nm.

### 4.5. Cell Uptake and Cell Viability Assay

#### 4.5.1. Release Profile and In Vitro Cytotoxicity

In order to measure the in-vitro drug release profile, 5 mL of IRI-Exos-15 were transferred into dialysis tubes with 3 KDa cut-off immersed in PBS at pH 7.4 or pH 4.2. The drug release study was performed at 37 °C and at different time intervals, up to 48 h.

The anti-tumor effect of engineered Exos loaded with IRI was evaluated by the standard MTT assay on U87-MG cells. Briefly, tumor cells (15 × 10^3^ cells/well) were seeded in 200 µL of media in a 96-well plate overnight. Tumor cells were treated with concentrations of Free-IRI and IRI-Exos-15 ranging between 0.25 and 10 µM for 24 and 48 h at 37 °C and 5% CO_2_. After incubation, CM was removed, and cells were incubated with MTT reagent for 3–4 h. Subsequently, 100 µL of DMSO was added to solubilize purple formazan crystals. Cytotoxic activity of Free-IRI and IRI-Exo-15 was then evaluated by standard MTT assay. Absorbance was measured by spectrophotometer at 545–630 nm. Survival rates were assessed compared to the negative control (wells containing only Untreated Exos). All experiments were repeated three times.

#### 4.5.2. Exos Uptake Flow Cytometry Study

1 × 10^5^ U87 MG cells/well were seeded in 48-well plates (Falcon^®^) and incubated for 24 h. Afterwards, cells were incubated with CM supplemented with IRI-Exos-15 or Free-IRI for 30 min, 2 h, 4 h, 6 h, 8 h, 24 h, and 48 h at a final concentration of 10 μM. Negative control consisted in the complete medium condition (medium, FBS, L-glutamine and penicillin/streptomycin) with an equal amount of PBS.

After different time points contacts, CM was removed, and the samples were washed three times with PBS (1x) to ensure particle removal from the outer cell membrane. Cells were then trypsinized for 5 min at 37 °C. After cell detachment confirmation at optical microscope, CM was added to neutralize the trypsin and all the content transferred to polystyrene round-bottomed tubes (Falcon^®^), before samples were immediately analyzed by flow cytometry. 

Every flow cytometry study was conducted in triplicate, and the average of the three samples was considered for forward scattering (FSC), side scattering (SSC) and fluorescence intensity mean in order to obtain reliable results in terms of viability and internalization information. All the analyses were conducted on BD FACSMelody^TM^.

Results are reported as the mean of the distribution of cell fluorescence intensity obtained by measuring 10,000 events averaged between three independent replicas. Error bars correspond to the standard deviation between the triplicates.

#### 4.5.3. Exos Confocal Microscopy Analysis

8 × 10^4^ U87 MG cells were seeded in 8-well μ-slide (inverted for high-end microscopy) (Ibidi^®^) and incubated at 37 °C for 24 h. Afterwards, cells were incubated for 24 h with IRI-Exos-15 previously stained with PKH67 according to the manufacturer’s protocol.

At different timepoints, CM was removed, and samples were washed three times with PBS (1x) and fixed with 4% paraformaldehyde (PFA) for 15 min. Cells were stored at 4 °C until confocal microscopy was performed. After fixation, cellular nucleus was stained with Hoechst dye 1:1000.

The sample was then observed using an TCS SP5 Confocal Laser Microscope (Leica Microsystems^©^).

Two lasers with different wavelengths were used for excitation of PKH67 and Hoechst dyes, respectively at 488 nm and 543 nm excitation and 500–530 nm and 560–610 nm emission wavelengths. HCX PL APO CS 63 × 1.40 Oil objective was used; laser intensities were between 5% and 20%.

## Data Availability

The data that support the findings of this study are available from the corresponding author upon reasonable request.

## Supplementary Materials

The following are available online at https://www.mdpi.com/article/10.3390/ijms22189896/s1.
