# Supplementary material for: A High Throughput Approach Based on Dynamic High Pressure for the Encapsulation of Active Compounds in Exosomes for Precision Medicine
Source: Int J Mol Sci. 2021 Sep 13;22(18):9896. doi: 10.3390/ijms22189896 (PMC8470411; doi:10.3390/ijms22189896)
Supplement: Supplementary file 1 [file ijms-22-09896-s001.zip › ijms-1347765-supplementary.pdf]

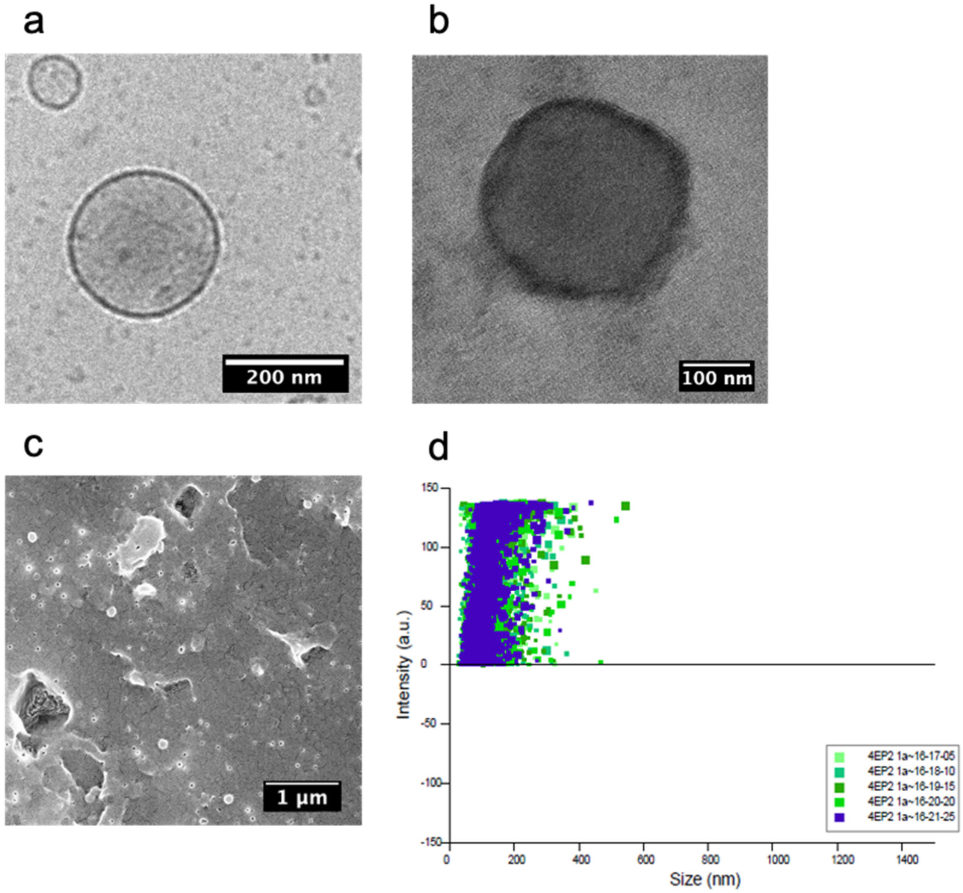

Figure S1. Characterization of untreated U87-Exos. (**a**) representative cryo-TEM, (**b**) TEM and (**c**) SEM images, show ovoidal-shaped vesicles, (**d**) Intensity/Size graph by Nanosight shows a size distribution in the range between 30-200 nm.

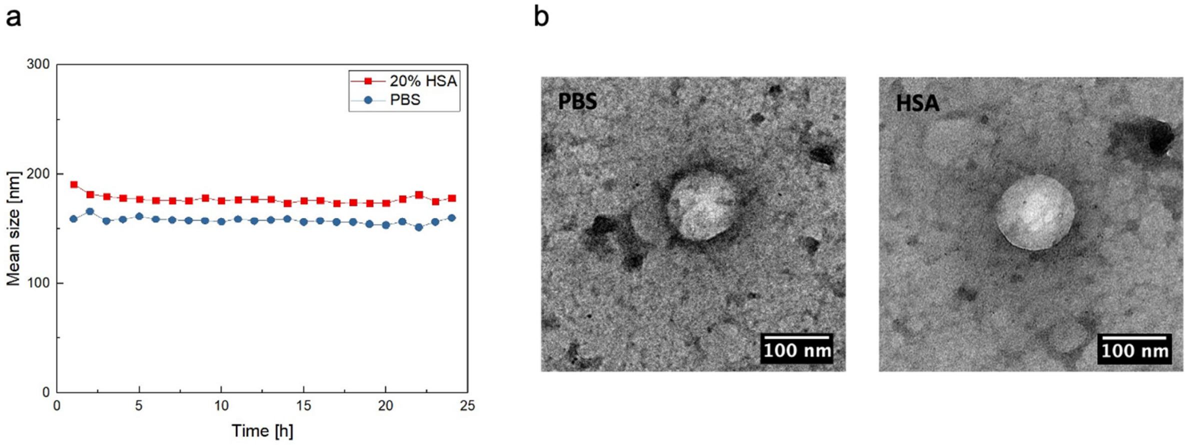

Figure S2. Stability studies of untreated U87-Exos in PBS and PBS containing 20% HSA at 37° C in a time lapse of 24 h. This analysis was performed through DLS, by setting the cell at 37 °C and measuring the size trend every hr to assess the vesicles biocompatibility. (**a**) Exos mean size is stable throughout the 24 h both in PBS and 20% HSA, (**b**) TEM observation after 24 h confirmed Exos stability.

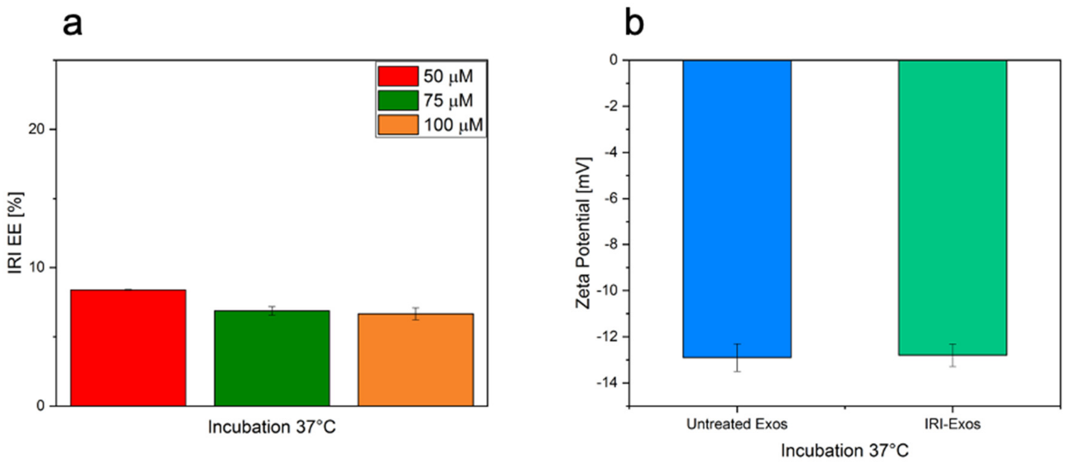

Figure S3. Co-incubation of Exos with IRI 2 hrs at 37 °C. (**a**) EE% of Exos at three different concentrations of IRI, (**b**) Zeta Potential comparison between Untreated Exos and Co-incubated vesicles shows no sensible difference in surface charge stability after interaction with drug.

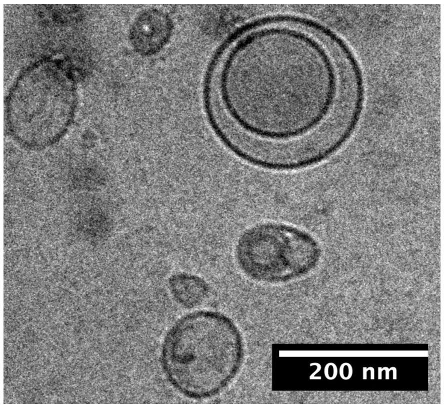

Figure S4. Representative Cryo-TEM image of IRI-Exos-15.
